# Cardamom (*Elettaria cardamomum* (L.) Maton) Seeds Intake Increases Energy Expenditure and Reduces Fat Mass in Mice by Modulating Neural Circuits That Regulate Adipose Tissue Lipolysis and Mitochondrial Oxidative Metabolism in Liver and Skeletal Muscle

**DOI:** 10.3390/ijms24043909

**Published:** 2023-02-15

**Authors:** Claudia Delgadillo-Puga, Ivan Torre-Villalvazo, Yonatan Y. Cariño-Cervantes, Cinthia García-Luna, Paulina Soberanes-Chávez, Patricia de Gortari, Lilia G. Noriega, Claudia J. Bautista, Luis Cisneros-Zevallos

**Affiliations:** 1 Departmento de Nutrición Animal Dr. Fernando Pérez-Gil Romo, Instituto Nacional de Ciencias Médicas y Nutrición Salvador Zubirán (INCMNSZ), Mexico City 14080, Mexico; 2 Departmento de Fisiología de la Nutrición, Instituto Nacional de Ciencias Médicas y Nutrición Salvador Zubirán (INCMNSZ), Mexico City 14080, Mexico; 3 Laboratorio de Neurofisiología Molecular, Instituto Nacional de Psiquiatría Ramón de la Fuente Muñiz, Mexico City 14370, Mexico; 4 Departamento de Biología de la Reproducción, Instituto Nacional de Ciencias Médicas y Nutrición Salvador Zubirán (INCMNSZ), Mexico City 14080, Mexico; 5 Department of Horticultural Sciences, Texas A&M University, College Station, TX 77843-2133, USA

**Keywords:** cardamom, energy expenditure, mitochondrial activity, fat mass, hypothalamic-pituitary-thyroid axis, hypothalamic-pituitary-adrenal axis, phenolic and terpenoid profiles

## Abstract

Cardamom seed (*Elettaria cardamomum* (L.) Maton; EC) is consumed in several countries worldwide and is considered a nutraceutical spice since it exerts antioxidant, anti-inflammatory, and metabolic activities. In obese individuals, EC intake also favors weight loss. However, the mechanism for these effects has not been studied. Here, we identified that EC modulates the neuroendocrine axis that regulates food intake, body weight, mitochondrial activity, and energy expenditure in mice. We fed C57BL/6 mice with diets containing 3%, 6%, or 12% EC or a control diet for 14 weeks. Mice fed the EC-containing diets gained less weight than control, despite slightly higher food intake. The lower final weight of EC-fed mice was due to lesser fat content but increased lean mass than control. EC intake increased lipolysis in subcutaneous adipose tissue, and reduced adipocyte size in subcutaneous, visceral, and brown adipose tissues. EC intake also prevented lipid droplet accumulation and increased mitochondrial content in skeletal muscle and liver. Accordingly, fasting and postprandial oxygen consumption, as well as fasting fat oxidation and postprandial glucose utilization were higher in mice fed with EC than in control. EC intake reduced proopiomelanocortin (POMC) mRNA content in the hypothalamic arcuate nucleus, without an impact on neuropeptide Y (NPY) mRNA. These neuropeptides control food intake but also influence the hypothalamic-pituitary-thyroid (HPT) and hypothalamic-pituitary-adrenal (HPA) axes. Thyrotropin-releasing hormone (TRH) mRNA expression in the hypothalamic paraventricular nucleus (PVN) and circulating triiodothyronine (T_3_) were lower in EC-fed mice than in control. This effect was linked with decreased circulating corticosterone and weight of adrenal glands. Our results indicate that EC modulates appetite, increases lipolysis in adipose tissue and mitochondrial oxidative metabolism in liver and skeletal muscle, leading to increased energy expenditure and lower body fat mass. These metabolic effects were ascribable to the modulation of the HPT and HPA axes. LC-MS profiling of EC found 11 phenolic compounds among which protocatechuic acid (23.8%), caffeic acid (21.06%) and syringic acid (29.25%) were the most abundant, while GC-MS profiling showed 16 terpenoids among which costunolide (68.11%), ambrial (5.3%) and cis-α-terpineol (7.99%) were identified. Extrapolation of mice-to-human EC intake was performed using the body surface area normalization equation which gave a conversion equivalent daily human intake dose of 76.9–308.4 mg bioactives for an adult of 60 kg that can be obtained from 14.5–58.3 g of cardamom seeds (18.5–74.2 g cardamom pods). These results support further exploration of EC as a coadjuvant in clinical practice.

## 1. Introduction

Cardamom (*Elettaria cardamomum* (L.) Maton; EC) seed is known as the ‘‘queen of spices’’ because of its delightful aroma, adaptable flavoring for a great variety of foods and beverages, and its purported beneficial health effects [1]. EC is considered a nutraceutical spice, since it contains several phenolic compounds and terpenoids that exert beneficial mood/behavioral and metabolic effects. Several studies have described the diverse health benefits of EC intake in humans and in rodents. EC intake improves the metabolism by reducing circulating levels of cholesterol [2], triglycerides and body mass index (BMI) [3]. In rats fed a high-carbohydrate high-fat diet (HCHF), EC intake increases glucose tolerance, improves insulin sensitivity, reduces oxidative stress and prevents liver inflammation [4]. Similar results are observed in diabetic rats and rats treated with dexamethasone [5,6,7]. Rats fed with an EC-containing diet also show a decreased atherogenic index; EC regulates blood pressure in both healthy and hypertensive rats [8,9], and prevents obesity [4]. Furthermore, an EC extract is able to increase HDL concentration [6,10].

Polyphenols and flavonoids present in EC exert high antioxidant capacity [11]. The neutralization of toxic radical oxygen species (ROS) prevents oxidative stress in tissues which, if allowed to accumulate, leads to accelerated aging, insulin resistance, cirrhosis, neurodegenerative diseases and cancer [12]. Individual EC compounds such as 1,8-cineole have been reported to decrease fat mass, vasodilation and to exert anti-inflammatory and antioxidant effects [13]. Previous studies on the brain confirm that EC intake shows protection against lipids peroxidation in cell membranes by activating the synthesis of antioxidant factors such as glutathione, catalase and superoxide dismutase, suggesting that the mechanism for the antioxidant effects of EC is the neutralization of ROS. Furthermore, EC also modulates intracellular signaling pathways, such as the acetylcholinesterase activity in hippocampus and cerebral cortex, improves memory and cognitive processes, reduces neuronal degeneration through stimulation of trophic factors such as the brain-derived neurotrophic factor (BDNF), and prevents amyloid ß protein associations in hippocampus and cortex, arresting the progression of Alzheimer’s disease [14].

Although several mechanisms have been proposed for the regulation of glycemia, dyslipidemia and oxidative stress by EC, there are no investigations to-date into its effects in the regulation of energy metabolism. As the hypothalamus-pituitary-thyroid (HPT) and hypothalamus-pituitary-adrenal (HPA) axes regulate fat mass, glucose tolerance and whole-body energy expenditure [15], the present study aims to gain insight into the endocrine and metabolic effects of EC intake on mice through the evaluation of hypothalamic peptides involved in energy balance and regulation of HPT and HPA axes that could help to understand the role of EC in energy homeostasis, body weight and food intake.

## 2. Results

### 2.1. Phenolics and Terpenoids Profiling by LC-MS and GC-MS

**Figure 1:**
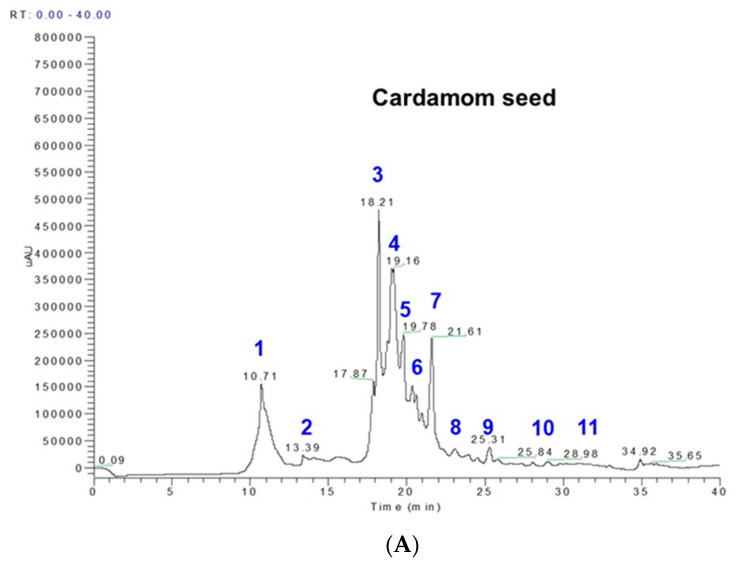

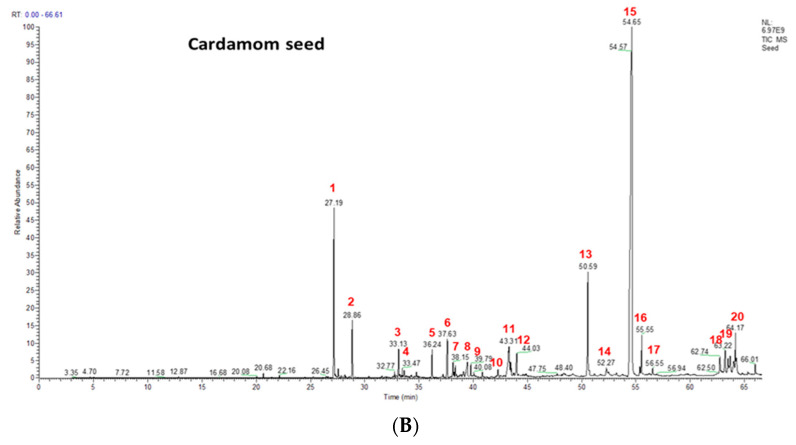
Phenolic and terpenoid profiles of cardamom seeds: (**A**) LC chromatograms of a methanolic extract of cardamom seed and peak assignment in blue of the identified phenolic compounds presented in Table 1; (**B**) GC-MS profiles of volatile compounds of cardamom seeds obtained by steam distillation and peak assignment in red of the identified volatile compounds presented in Table 2.

Cardamom seed phenolics and terpenoids characterization was done by LC-MS and GC-MS analysis, respectively. The detailed study of cardamom seed and previous reports on phenolics shows the identification of protocatechuic acid, gentisic acid, caffeic acid, vanillic acid, p-coumaric acid and ferulic acid [16] from cardamom. In our study, we found 11 phenolic compounds among which protocatechuic acid (23.8%), caffeic acid (21.06%) and syringic acid (29.25%) were the major phenolics identified in cardamom seed. Furthermore, syringic acid, 5-O-caffeoylquinic acid, sinapoylquinic acid, feruloyl quinic acid and rutin are reported for the first time in EC seeds (Figure 1A, Table 1). The volatile chemistry of essential oils from cardamom seed by GC-MS analysis gave the identification of 16 terpenoids among which costunolide (68.11%), ambrial (5.3%) and cis-α-terpineol (7.99%) were the major terpenoids identified in cardamom seed. On the other hand, α-acorenol, cubebol, isopathulenol, globulol, trans-farnesol, ambrial, eicosane, costunolide, coronarin and erucylamide were some of the mono and diterpenes, sesquiterpenes and alkanes reported for the first time in cardamom essential oil studies. We also report fatty acid esters of palmitic and stearic acid as β-mono palmitin and β-mono stearin, the fatty acid amide erucylamide and triterpene β-sitosterol (Figure 1B, Table 2). In the literature, the Indian variety of cardamom had the major compounds characterized as 1,8-cineole (38.7%), β-pinene (13.6%), α-terpineol (12.6%), spathulenol, (8.3%), 4-terpineol (4.5%), germacrene-D (3.0%), α-pinene (2.8%) and β-selinene (2.7%) [17]. In general, the amount of phenolics (chlorogenic acid equivalent) and essential oil content (terpenoids) in EC was 124.53 mg phenolics/100 g seeds and 404 mg essential oil/100 g seeds, respectively. Thus, the total amount of bioactive compounds (phenolics + terpenoids) in EC is ~528.53 mg bioactives/100 g seeds.

### 2.2. Cardamom Intake Reduces Fat Mass Accretion in Mice

**Figure 2:**
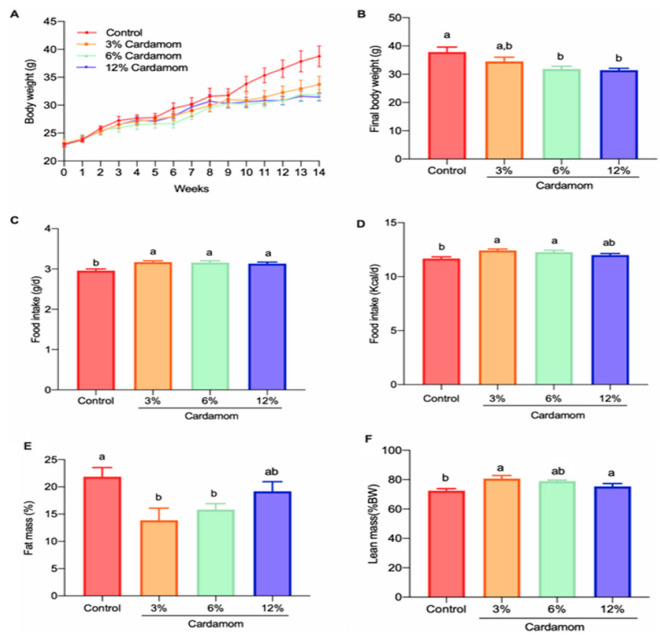
Body weight gain, food intake and body composition of mice fed a control diet or a diet containing 3%, 6% or 12% of *Elettaria cardamomum* (EC). (**A**) body weight in grams from 0–14 weeks one way repeated measures ANOVA; (**B**) final body weight in grams at week 14; (**C**) mean daily food intake; (**D**) mean daily energy intake in calories; (**E**) percentage of fat mass; and (**F**) lean mass of mice fed with control diet or a diet containing 3%, 6% or 12% cardamom. Data are presented as the mean ± S.E.M., *n* = 8 mice per group. The differences were considered statistically significant when *p* < 0.05. Mean values with different lowercase letters show statistical differences between each other.

To evaluate the metabolic and endocrine effects of EC, we fed mice with a control diet or isoenergetic diets containing 3%, 6% or 12% powdered EC for 14 weeks. Body weight gain was lower in mice fed with EC than in the control group from week 10 until the end of the study (Figure 2A,B). Unexpectedly, food intake (expressed as grams or calories) was slightly, but significantly, higher in mice fed EC than in mice fed control diet (Figure 2C,D). To determine if the differences in body weight between groups were due to a variance in muscle mass or adipose tissue content, we evaluated body composition using magnetic resonance. Figure 2E,F shows that EC intake reduced fat mass but maintained or slightly increased lean body mass. These results show that EC intake attenuates body weight gain by preventing fat mass accretion.

### 2.3. Mice Fed with Diets Containing Cardamom Seeds Display Reduced Adipocytes Size Associated with Increased Lipolysis

**Figure 3:**
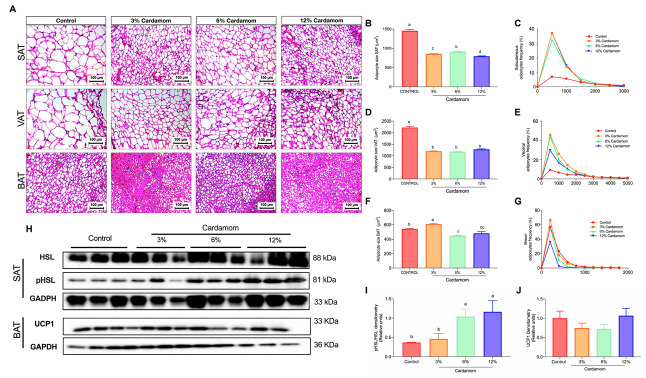
Adipose tissues morphology, histological analyses, UCP-1 and HSL protein content of mice fed a control diet or a diet containing 3%, 6% or 12% of *Elettaria cardamomum* (EC). (**A**) hematoxylin and eosin staining of subcutaneous (SAT), visceral (VAT) and brown adipose tissues (BAT); (**B**) SAT mean adipocyte size; (**C**) SAT adipocyte frequency distribution; (**D**) VAT mean adipocyte size; (**E**) VAT adipocyte frequency distribution; (**F**) BAT lipid droplets frequency distribution; (**G**) BAT lipid droplets frequency distribution; (**H**) WB images of total HSL, phospho HSL and GAPDH content in SAT, and UCP-1 and GAPDH in BAT; (**I**) phospho-HSL/total HSL protein abundance ratio; and (**J**) UCP1/GAPDH protein abundance ratio of mice fed a control diet or a diet containing 3%, 6% or 12% EC. Data are expressed as the mean ± S.E.M., *n* = 8 mice per group. The differences were considered statistically significant when *p* < 0.05. Mean values with different lowercase letters show statistical differences between each other. Digital photographs were taken from each section at 20×.

To gain insight into the mechanisms involved in the reduction of fat mass in mice fed EC, we analyzed the morphological features of subcutaneous, visceral and brown adipose tissues (SAT, VAT, and BAT, respectively). Mice fed with EC reduced adipocyte size in SAT and VAT in comparison to the control (Figure 3A,B,D). Frequency distribution analysis showed that 60% of adipocytes in SAT and VAT of EC fed mice were smaller than 1500 μm, compared to ≈10% in adipocytes of the control diet (Figure 3C,E). To evaluate if the reduction in adipocyte size of EC fed mice was due to increased lipolysis, we determined hormone-sensitive lipase (HSL) abundance by evaluating HSL content and its phosphorylated form. Mice fed 6% and 12% EC showed an increase in HSL phosphorylation, suggesting increased lipolytic activity. Similarly, we found that 6% and 12% EC reduced BAT adipocytes size with respect to the other groups (Figure 3F,G). However, we did not find differences in UCP-1 protein content (Figure 3H,J). These results indicated that EC intake might be stimulating HSL activity in white adipose tissues, increasing triglyceride hydrolysis and consequently reducing lipids content in adipocytes. By contrast, EC did not increase BAT thermogenic activity at any of the doses tested.

### 2.4. Cardamom Seeds Intake Increases Mitochondrial Activity and AMPK Content in Skeletal Muscle

**Figure 4:**
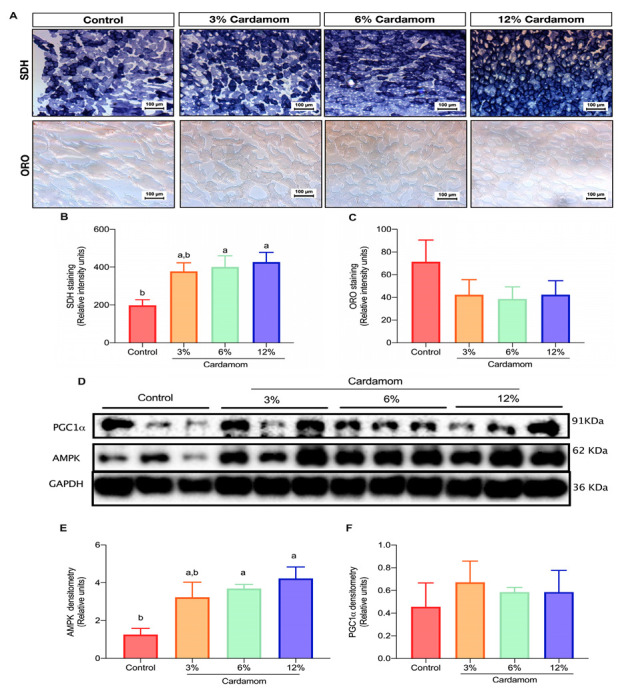
Skeletal muscle mitochondrial activity, lipid content, PGC1α and AMPK protein density of mice fed a control diet or a diet containing 3%, 6% or 12% of *Elettaria cardamomum* (EC). (**A**) representative photomicrographs of succinate dehydrogenase (SDH) and oil red O (ORO) staining in the skeletal muscle; (**B**) SDH staining densitometric analysis; (**C**) ORO staining densitometric analysis; (**D**) representative image of PGC-1α and AMPK immunoblot; (**E**) densitometric analysis of AMPK/GADPH protein abundance ratio; and (**F**) densitometric analysis of PGC1α/GADPH protein abundance ratio of mice fed a control diet or diets containing 3%, 6% or 12% EC. Results are presented as the mean ± S.E.M., *n* = 8 mice per group. The differences were considered statistically significant when *p* < 0.05. Mean values with different lowercase letters show statistical differences between each other. Digital photographs were taken from each section at 20×.

Increased adipose tissue lipolysis augments free fatty acid release into circulation and hence, fatty acid uptake in metabolic tissues such as skeletal muscle and liver. Excessive accumulation of fatty acids in skeletal muscle fibers leads to mitochondrial dysfunction and consequently, to the development of metabolic alterations [18]. Since EC increased adipose tissue lipolysis, we investigated mitochondrial activity and lipids content in skeletal muscle of mice fed with control or EC diets. Figure 4A,C shows a non-significant tendency of lipid droplets to decrease in the skeletal muscle of EC-fed mice with respect to the control group. Since in the present study, diets offered to animals were based on the AIN93 formulation [19], mice ingested the recommended fat to be in optimal health. Therefore, the lipids content in myofibers was expected to be low in all groups. However, since EC-fed mice had increased HSL activity in adipose tissue, it was expected a high fat accumulation in skeletal muscle. Interestingly, this was not the case-suggesting that the increased mitochondrial oxidative capacity of skeletal muscle of EC fed mice prevented intramyocellular lipid accumulation. Furthermore, Figure 4A,B shows a significant increase in SDH activity in the skeletal muscle of EC fed mice when compared to the control. To define whether the observed differences in mitochondrial activity between groups were mediated by increased PGC-1α or AMPK content, we determined the amount of those proteins by immunoblotting. Unexpectedly, we did not observe significant differences in PGC-1α protein content in the skeletal muscle of EC fed mice. However, AMPK content was higher in all groups of EC fed mice when compared to the control (Figure 4D–F). These results indicated that EC intake increases mitochondrial activity by activating a mechanism associated with increased AMPK content. Consequently, EC fed mice presented an increased mitochondrial oxidative capacity that prevented accumulation of lipids in their skeletal muscle.

### 2.5. Mice Fed with Diets Containing Cardamom Seeds Showed High Hepatic Mitochondrial Activity

**Figure 5:**
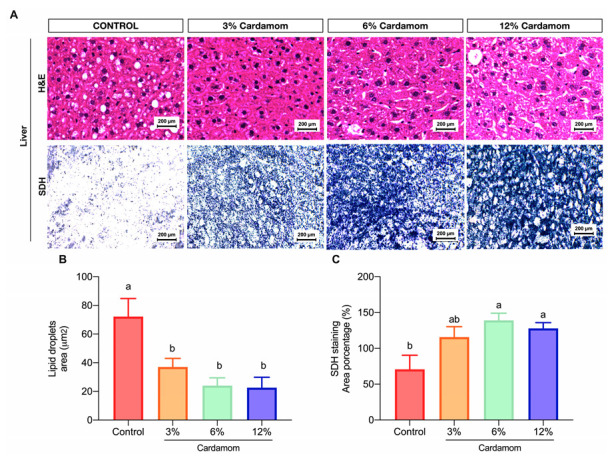
Liver morphology and hepatic SDH activity of mice fed a control diet or a diet containing 3%, 6% or 12% of *Elettaria cardamomum* (EC). (**A**) representative photomicrographs were taken from each section at 40× of hematoxylin and eosin (H&E) and succinate dehydrogenase (SDH) staining in the liver; (**B**) lipid droplets’ mean area; and (**C**) SDH densitometry as (%) of liver sections of mice fed a control diet or a diet containing 3%, 6% or 12% EC. Results are presented as the mean ± S.E.M., *n* = 8 mice per group. The differences were considered statistically significant when *p* < 0.05. Mean values with different lowercase letters show statistical differences between each other. Digital photographs were taken from each section at 40×.

Similar to skeletal muscle, the liver is another organ with a high capacity to clear fatty acids from circulation. To evaluate hepatic lipids content and mitochondrial activity in EC groups, we analyzed the content in lipid droplets and the SDH activity in liver sections of all groups. Figure 5A,B shows that the size of lipids droplets was lower in EC mice than in control. Interestingly, SDH activity was higher in EC-fed mice (Figure 5A,C), suggesting that EC intake increased the mitochondrial activity in the liver also, which might prevent excessive hepatic lipid accumulation.

### 2.6. Cardamom Intake Increased Oxygen Consumption and Metabolic Flexibility in Mice

**Figure 6:**
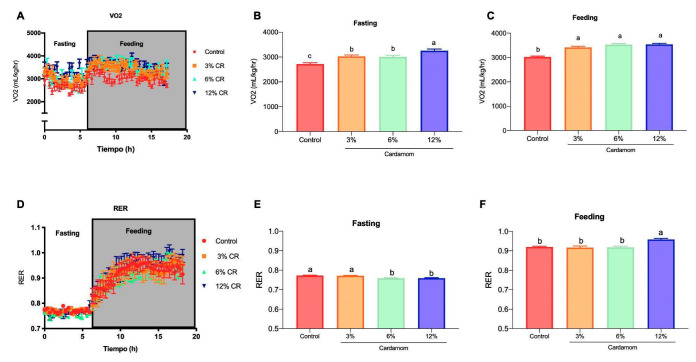
Energy expenditure and substrate oxidation of mice fed a control diet or a diet containing 3%, 6% or 12% of *Elettaria cardamomum* (EC). (**A**) Oxygen consumption (VO_2_) during fasting and feeding periods determined by indirect calorimetry analysis; clear and shaded zones indicate fasting and feeding periods, respectively. Average oxygen consumption during (**B**) fasting and (**C**) feeding. (**D**) Respiratory exchange ratio (RER) and average RER during (**E**) fasting and (**F**) feeding periods of mice fed a control diet or a diet containing 3%, 6% or 12% EC. Data are presented as the mean ± S.E.M., *n* = 8 mice per group. The differences were considered statistically significant when *p* < 0.05. Mean values with different lowercase letters show statistical differences between each other.

To evaluate if the increased mitochondrial activity in skeletal muscle and liver of EC fed group of mice can be observed in an augmented whole-body oxygen consumption, mice were transferred to metabolic cages with an open-flow gas-exchange system to measure oxygen consumption and CO_2_ production during feeding and fasting states. As expected, EC fed mice presented higher oxygen consumption than controls during both feeding and fasting periods (Figure 6A–C). Indirect calorimetry also allowed us to determine the proportion of substrates that were oxidized during fasting and feeding periods [20]. As observed in Figure 6D, during fasting, the respiratory exchange ratio (RER) of all mice was below 0.8, indicating exclusive fat oxidation; whereas during the feeding period, RER increased to almost 1, attributed exclusively to glucose utilization as energy substrate. However, RER of EC groups was lower and higher than controls during fasting and feeding periods, respectively (Figure 6E,F). These results revealed that EC fed mice had higher fat oxidative capacity during fasting and an enhanced glucose tolerance during feeding than control, showing an adequate switching from fat to glucose oxidation in the fasting-to-feeding transition, which is known as metabolic flexibility and is the hallmark of metabolic health [21].

### 2.7. Cardamom Intake Modulated the Expression of Hypothalamic Peptides and the Circulating Levels of Triiodothyronine (T3) and Corticosterone

**Figure 7:**
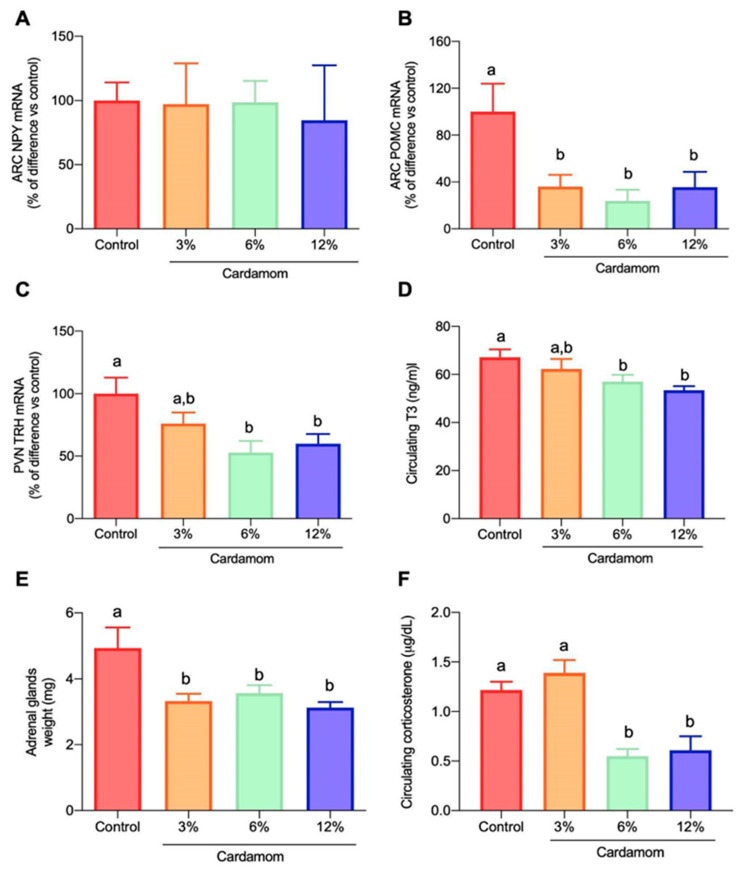
Hypothalamic peptide gene expression and circulating triiodothyronine (T_3_) and corticosterone concentration of mice fed a control diet or a diet containing 3%, 6% or 12% of *Elettaria cardamomum* (EC). (**A**) ARC NPY mRNA and (**B**) ARC POMC mRNA content, (**C**) PVN TRH mRNA expression, (**D**) circulating T_3_ levels, (**E**) adrenal glands’ weight and (**F**) circulating corticosterone content in mice fed a control diet or a diet containing different concentrations of EC (3%, 6% or 12%) for 14 weeks. Results are presented as the mean ± S.E.M., *n* = 4–8 mice per group. Mean values with different lowercase letters show statistical differences between each other. One-way ANOVA showed a significant effect of diet: (**B**) F_(3,15)_ = 5.218, *p* < 0.05, (**C**) F_(3,26)_ = 4.398, *p* < 0.05, and (**E**) F_(3,22)_ = 6.335, *p* < 0.01.

To gain insight into the mechanisms through which cardamom intake influences energy metabolism in adipose tissues, skeletal muscle and liver, we evaluated the mRNA expression of central peptides involved in the regulation of energy balance. We found that EC-fed mice had similar NPY but lower POMC mRNA expression in the hypothalamic ARC (Figure 7A,B). Mice fed 6% or 12% EC had lower PVN TRH mRNA content than control mice (Figure 7C) and reduced circulating T_3_ levels (Figure 7D). We also evaluated weight of adrenal glands and circulating corticosterone concentration. Diets with 3%, 6% and 12% concentration of EC induced a decrease of 32%, 26% and 36%, respectively, of adrenal glands’ weight when compared to control (100%, 4.9 mg) (Figure 7E). As expected, plasma corticosterone levels were lower in mice fed 6% and 12% EC than in the other groups (Figure 7F). These results revealed a central mechanism of action of EC, regulating the expression of the peptides that control food intake and energy expenditure and as a consequence, a fine tuning of the functioning of HPT and HPA axes.

## 3. Discussion

**Figure 8:**
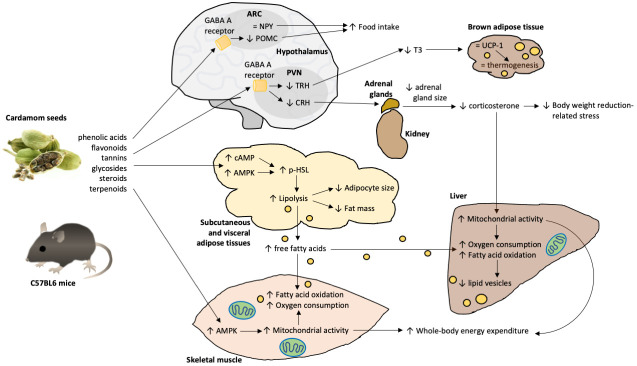
Scenario 1: Parallel central and peripheral effects of cardamom intake regulating energy metabolism. The different compounds present in cardamom seeds exerted central and peripheral effects modulating energy balance in mice. Central effects include the modulation of NPY and POMC expression, the hypothalamic peptides controlling food intake and energy expenditure, probably by activation of the GABA_A_ receptor. The activity of the TRH and CRH neurons in the paraventricular nucleus (PVN) are also modulated by GABA_A_ receptors. These neurons regulate the circulating levels of triiodothyronine (T_3_) and corticosterone. The metabolic activities of T_3_ in peripheral tissues include the activation of uncoupling protein 1 (UCP-1) mediated thermogenesis in brown adipose tissue. Cardamom seeds also exerted peripheral effects in adipose tissue, skeletal muscle and the liver, in part by increasing AMP-activated protein kinase (AMPK). AMPK signaling in adipose tissue increased lipolysis by activation of hormone sensitive lipase (HSL). In skeletal muscle and liver, AMPK increases oxidative capacity by increasing mitochondrial biogenesis and activity. Conversely, chronic corticosterone signaling impairs mitochondrial biogenesis and function. Thus, the reduction in circulating corticosterone by cardamom consumption favors oxidative metabolism in metabolic tissues, reducing body fat by increasing lipolysis and whole-body energy expenditure through augmented mitochondrial activity in skeletal muscle and liver. Upwards arrow = upregulation, downwards arrow = downregulation.

**Figure 9:**
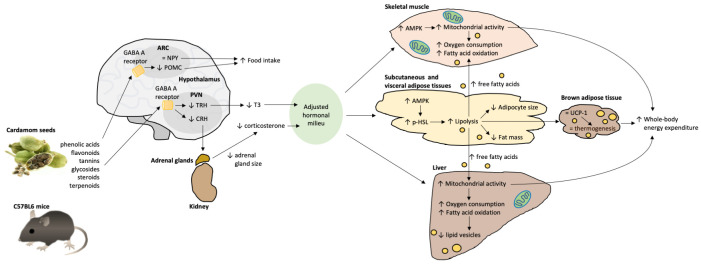
Scenario 2: Modulation of the thyroid and adrenal axes by cardamom intake, generating an “adjusted hormonal milieu” with peripheral effects regulating energy metabolism. Cardamom seed consumption modulates the expression of NPY and POMC, the hypothalamic peptides controlling food intake and energy expenditure, probably by activation of the GABA_A_ receptor in the arcuate nucleus (ARC). Furthermore, activation of the GABA_A_ receptor in the paraventricular nucleus (PVN) influences TRH and CRH neurons which regulates the synthesis and secretion of triiodothyronine (T_3_) and corticosterone. The metabolic activities of T_3_ in peripheral tissues includes the stimulation of lipolysis in adipose tissue through activation of hormone sensitive lipase (HSL), the activation of uncoupling protein 1 (UCP-1) mediated thermogenesis in brown adipose tissue, and the stimulation of oxidative capacity in liver and skeletal muscle by increasing mitochondrial biogenesis and activity, in part by increasing AMP-activated protein kinase (AMPK). Conversely, corticosterone down-regulates some metabolic pathways that are activated by T_3_. Thus, the modulation of PVN activity mediated by cardamom intake could reduce T_3_ secretion but also corticosterone synthesis, increasing some of the thyroid-mediated metabolic actions in peripheral tissues. The regulation of the hypothalamic-pituitary-thyroid (HPT) and hypothalamic-pituitary-adrenal (HPA) axes by cardamom intake generates an “adjusted hormonal milieu”, reducing body fat by increased lipolysis and increasing oxygen consumption and energy expenditure through augmented mitochondrial activity in skeletal muscle and liver. Upwards arrow = upregulation, downwards arrow = downregulation.

In the present study, we described central and peripheral effects of cardamom seed ingestion that modulates energy balance in mice. The central effects of EC intake includes the regulation of the expression of hypothalamic peptides involved in the control of food intake, energy expenditure and the modulation of the HPT and HPA axes. The peripheral effects consist of the activation of lipolysis in adipose tissue and augmented mitochondrial activity in skeletal muscle and liver. The combined central and peripheral actions of EC result in increased energy expenditure and fat oxidation leading to lower fat mass. The results of the present study can be interpreted in two main ways: in the first, the effects of EC are both central and peripheral parallel activities (Scenario 1, Figure 8), and in the other, the main actions of EC are via the modulation of the central thyroid and adrenal axes, generating an “adjusted hormonal milieu” with peripheral activities (Scenario 2, Figure 9).

The present results show that the intake of EC seeds reduces body weight gain by a reduction in fat mass. This observation agrees with the reported decrease in body weight gain of rats fed a HCHF diet containing 1% cardamom powder [4]. The reduction in fat mass was likely due to enhanced mobilization of lipid stores and increased energy expenditure in metabolic organs, increased hormone-sensitive lipase activity in adipose tissue, as determined by increased activation of Ser563 phosphorylation. Activation of HSL in adipose tissue is mediated by the AMPK and PKA signaling pathways [22]. Thus, in the first proposed scenario, the mechanism involved in the cardamom-induced increase in HSL activity can include the activation of AMPK by the cardamom terpenes or flavonoids (Table 1 and Table 2) in adipose tissue as it has been demonstrated with Rosemary (*Salvia rosmarinus*) [23], Saffron (*Crocus sativus* Linn.) [24] and soy-derived geinstein [25]. Other mechanisms may include the activation of PKA by a diterpene-induced increase in cAMP content [26] (Figure 8).

Free fatty acids released from adipose tissue are readily absorbed by metabolic tissues, such as the skeletal muscle and liver. The increased protein expression of HSL-Ser563 phosphorylated form in adipose tissue of cardamom-fed mice increased the release of free fatty acids, and their uptake by metabolic tissues such as skeletal muscle and liver [27]. Interestingly, neither the skeletal muscles nor the liver of the cardamom-fed mice showed greater lipid accumulation. Instead, they showed lower lipid content associated with higher mitochondrial activity, as determined by SDH activity. Accordingly, EC-fed mice presented increased whole-body oxygen consumption and an enhanced metabolic flexibility as assessed by the respiratory exchange ratio (RER) [27]. Metabolic flexibility refers to the physiological switch between fatty acids/glucose utilization during a fast-to-feeding transition [27]. Thus, those results are evidence of improved mitochondrial function in cardamom-fed groups.

Energy homeostasis is regulated by the interaction between central and peripheral signals. The hypothalamus is the main central region that integrates peripheral signals related to energy homeostasis. These signals coordinate the activity of the different hypothalamic nuclei that synthesize orexigenic or anorexigenic peptides to induce or inhibit food intake and energy expenditure, respectively. The reduction in the markers of the HPT axis in the EC fed mice was probably a response to decreased body weight. Weight loss leads to a decrease in T_3_ release from the thyroid in the circulation [28], which in turn, is unable to activate the synthesis of hypothalamic TRH or pituitary TSH, as a negative energy balance (NEB)-induced blockade of the negative feedback mechanism of the HPT axis. The NEB-induced blockade of the negative feedback mechanism results from an increased activity of the type 2-deiodinase in the mediobasal hypothalamus that converts T_4_ into T_3_, thus increasing hypothalamic T_3_ levels and repressing the transcription of TRH gene in the PVN [29]. It remains to be elucidated whether cardamom is able to stimulate type 2 deiodinase activity. In conditions of energy homeostasis (when animals maintain their body weight), in contrast, low levels of T_3_ increase the transcription rate of TRH gene in the PVN, and promote TRH release into the portal blood that activates TSH release and finally recovers T_3_ blood concentration and returns the HPT axis to its basal functioning. In this study, the blockade of the negative feedback mechanism observed in EC-fed groups seemed to result as a compensating change to the greater degradation of lipids in the adipose tissue, which avoided the activation of thermogenesis in the brown adipose tissue, mainly regulated by high T_3_ levels [30,31,32]. The low T_3_ levels could be responsible for the lack of increase in BAT UCP1 content, therefore the active thermogenesis in EC-fed mice should be due to effectors other than T_3_. Moreover, as occurs in other NEB conditions such as fasting or malnutrition, the body weight loss of EC-fed mice was able to modulate the expression of hypothalamic feeding-regulatory peptides in such a way that the animals increased their food intake due in part to the resulting reduced levels of peptides with anorectic actions such as POMC in the ARC and TRH in the PVN [33,34,35].

Interestingly, corticosterone plasma levels did not increase in the EC-fed group despite their body weight loss, as it is a stressful stimulus. As sustained elevations in serum corticosterone induce animals and humans’ anxious behavior, its low circulating concentration might be a direct effect of cardamom intake in the hypothalamus. It has been shown that the EC compound quercetin inhibits CRH mRNA expression in the PVN through the activation of GABA_A_ receptors and has anxiolytic effects [36,37,38]. Moreover, the increased food intake of EC-fed mice could not be due to the hyperphagic effects of corticosterone. This is supported by the unchanged NPY mRNA levels found in the EC-fed mice, which increases when corticosterone concentration elevates [37]. Another possibility for the decreased ARC POMC and PVN TRH mRNA expression in mice fed with cardamom seeds is through direct actions on those neurons. Quercetin, an antioxidant compound present in EC, seems to act through GABA_A_ receptors since the i.p. administration of bicuculline (GABA_A_ receptor antagonist) blocked the antinociceptive effect of quercetin [39]. Moreover, PVN TRH and ARC POMC neurons receive inhibitory GABAergic inputs [35,40,41,42], and GABA_A_ receptor is expressed in PVN and ARC neurons [43]; thus, it could be possible that EC exerts its effects directly on hypothalamic PVN and ARC neurons reducing TRH and POMC mRNA transcription. This hypothesis warrants further investigation. Furthermore, it has been recently revealed in mice that stress increases ARC POMC expression and is associated with reduced food intake [21]. Thus, the reduction in POMC mRNA expression in ARC is probably one of the mechanisms through which cardamom intake exerts its beneficial metabolic activities. All these findings support our hypothesis that a cardamom-based diet stimulates appetite by acting on arcuate anorexigenic peptide expression, and by reducing the mRNA levels of POMC and TRH which also has anorectic effects [33]. Interestingly, the high lipid degradation observed in those animals prevented their body weight from increasing even when they increased their food intake.

Glucocorticoids and thyroid hormone signaling in target tissues are regulated by an intricate crosstalk, where the outcome depends on the concentration and activity of each hormone [44]. Some activities are synergistically activated, whilst others are mutually excluding [45,46]. This is the case of mitochondrial biogenesis and function, where mitochondrial dynamics and activity is increased by T_3_ but impaired by continuous corticosterone exposure [47,48,49]. In the proposed second scenario, the increase in mitochondrial activity in liver and skeletal muscle in cardamom-fed mice is due to an “adjusted hormonal milieu” where T_3_ is low, but so is corticosterone, allowing proper signaling of the T_3_ in metabolic tissues (Figure 9). Future studies are warranted to explore these scenarios.

This study aimed to evaluate the metabolic effects of a nutraceutical spice in mice, in order to assess its therapeutic potential to be used as a non-pharmacological therapy for metabolic disorders. We postulated two possible scenarios of how EC modulates metabolic tissues in order to fine-tune energy balance. However, more studies are needed to unravel the intricate mechanisms through which a nutraceutical spice exerts its metabolic effects. Since EC seeds contain a complex matrix of phytochemicals with potential health effects, we did not expect a single molecule to be responsible for its metabolic actions. Thus, we did not include a positive control, i.e., a group of rats fed with a diet supplemented with a single EC-derived compound as we have done in previous studies [50,51]. We evaluated three different doses of EC seeds to evaluate its dose-dependent effects. Interestingly, many biochemical and molecular parameters were modulated by EC in all the doses tested, indicating that EC can exert beneficial metabolic effects when included in a diet as low as 3%.

To obtain a reference dose for future clinical studies, the body surface area (BSA) normalization method can be used to convert the dose information for mice to equivalent human intake [52,53]. For instance, in the present study, the consumption of a 3 g/day of feed containing cardamom seeds in three dose content (3%, 6% and 12%) in a diet provided the mice with 0.47, 0.95 and 1.90 mg/day of bioactive compounds (phenolics + terpenoids), respectively (Figure 2C and the table in Section 4.5). This amount of bioactive compounds for mice of ~30-g average body weight corresponds to 15.8, 31.7 and 63.4 mg bioactives/kg per day, which multiplied by the animal Km factor (3) and divided by the human Km factor (37) will give a human dose equivalent of 1.28, 2.57 and 5.14 mg bioactives/kg per day, respectively. The Km factor is obtained by dividing body weight (kg) and BSA (m^2^) and used for drug dose conversion between mg/kg and mg/m^2^, and its value for different animal species is available in the literature [52]. Accordingly, for an average human adult of 60 kg, the intake of bioactive compounds from three doses of cardamom seeds (3%, 6% and 12%) would correspond to ~76.98, 154.2 and 308.4 mg bioactives/day, respectively. Thus, considering that cardamom seeds contain ~528.53 mg bioactives/100 g seeds, then the calculated intake of cardamom for the three doses (3%, 6% and 12%) would correspond to 14.5, 29.1 and 58.3 g of cardamom seeds, respectively. This amount of cardamom seeds could be supplied by daily use of 18.5, 37.1 and 74.2 g cardamom pods, respectively (1 g cardamom pod supplies ~0.78 g seeds) or alternatively, the daily cardamom seed intake could also be supplied by extracting the bioactive compounds in the form of a dietary supplement.

## 4. Materials and Methods

### 4.1. Cardamom Samples

The EC seeds were harvested in Guatemala and kindly supplied by Heifer International. We used methanolic extraction for non-volatile polyphenols, while for volatile terpenoids we used steam distillation to obtain the essential oils of cardamom. The profiles of the non-volatile polyphenol were evaluated by liquid chromatography coupled to mass spectrometry (LC-MS). The profiles of volatile terpenoids were determined by gas chromatography coupled to mass spectrometry (GC-MS).

### 4.2. Sample Preparation for Polyphenol Analysis and LC-MS Polyphenol Profiling

The grounded powder of *Elettaria cardamomum* EC (~1 g) was dissolved in methanol (10 mL) and stirred for 15 h at 4 °C. The extract was then centrifuged and concentrated at 4000 rpm (2147× *g*) at 45 °C until all of the volatile solvents were evaporated in a Centrivap concentrator connected to a cold trap (Labconco, Kansas City, MO, USA), obtaining a yield extract of 126.2 mg. The extracts obtained from the EC were re-dissolved in 10 mL methanol and the appropriate amount of extract was taken (as given above) to make a concentration of ~5 mg/mL and filtered with a 0.22 µm PTFE filter (Agilent, Santa Clara, CA, USA); and a volume of 10 µL was injected into the LC-MS in 3 replicates. The determination of individual phenolic compounds was performed on a Surveyor HPLC/MS system equipped with an autosampler, a Surveyor 2000 quaternary pump, and a Surveyor UV 2000 PDA detector using a C_18_ reverse phase (150 mm × 4.6 mm, Atlantis, Waters, Ireland; particle size = 5 μm) column connected to a LCQ Deca XP Max MSn system (Thermo Finnigan, San Jose, CA, USA) with a Z-spray ESI source run by Xcalibur software, version 1.3 (Thermo Finnigan-Surveyor, San Jose, CA, USA). The mobile phase flow rate was set at 0.25 mL/min, while the elution gradients were performed with solvent A, consisting of acetonitrile/methanol (1:1) (containing 0.5% formic acid); and solvent B, consisting of water (containing 0.5% formic acid). The applied elution conditions were: 0–2 min, 2% A, 98% B; 3–5 min, 5% A, 95% B; 5–7 min, 25% A, 75% B; 7–12 min, 55% A, 45% B; 12–24 min, 55–80% A; 24–27 min held isocratic at 80% A, 28–30 min 90% A, 10% B; 31–33 min held isocratic, 100% A; and 34–40 min, 2% A, 98% B, to the starting condition. The chromatograms were monitored at 280 nm, and complete spectral data were recorded in the range 200–600 nm. ESI was performed in the negative ionization mode, nitrogen was used as sheath gas with a flow of 59 arbitrary units, and He gas was used as dampening gas. The capillary voltage was −4.17 V; spray voltage, 5 kV; capillary temperature, 275 °C; and tube lens voltage was set at −55 V. Collision energies of 30% were used for the MSn analysis. Phenolic quantification—using PDA chromatogram area peaks and a standard curve of chlorogenic acid—are reported as mg chlorogenic acid equivalents per 100 g seeds per individual peak, and the sum as total phenolics.

### 4.3. Sample Preparation for Terpenoid Analysis and GC-MS Profiling

Essential oil was extracted by taking ~5 g of sample to which 500 mL of distilled water was added and subjected to steam distillation to obtain the essential oils with a yield of ~20.2 mg. The oil obtained was dried over anhydrous sodium sulfate and stored in amber and air-tight sealed vials at 0 °C until analyzed and tested.

GC-FID analyses were carried out on trace GC Ultra gas chromatograph equipped with an auto sampler with a RX-5MS capillary column (60 m × 0.25 mm, 0.25 μm film thickness) which was coupled to a mass spectrometer (Thermo Scientific, WestPalm, FL, USA). The column temperature was programmed to rise from 50 to 280 °C at a rate of 3 °C/min. The carrier gas was helium with a flow rate of 1.5 mL/min. Scan time and mass range were 1 s and 50–550 *m*/*z*, respectively, and the acquired data were processed using Xcalibur software (Thermo Fisher Scientific, version 2.0.7). The peaks obtained were tentatively identified by comparing their mass spectra with those included in the NIST-05a. The essential oils were injected at a concentration of 2.0 mg/mL.

### 4.4. Animals

Male C57BL/6 mice of 5 weeks of age and weighing 20–25 g (*n* = 32) were obtained from the Experimental Research Department and Animal Care Facility at the Instituto Nacional de Ciencias Médicas y Nutrición Salvador Zubirán (DIEB-INCMNSZ) and housed in micro isolation cages (3 mice per cage) with controlled temperature at 23 °C and a 12-h on/12-h off light-dark cycle (7:00 a.m.–7:00 p.m.). Animals were fed a diet with or without cardamom seeds as described in Section 2.5. All animal procedures were conducted in accordance with the recommendations and procedures from the Guide for Care and Use of Laboratory Animals of the Institute for Laboratory Animal Research. The Animal Care Committee of the Instituto Nacional de Ciencias Médicas y Nutrición Salvador Zubirán (CINVA-INCMNSZ. Ref. NAN-1992-20-21-1) approved the study.

### 4.5. Experimental Diets

Animals (32 mice total) were randomly assigned into four groups (*n* = 8 per group) respectively receiving a control diet or a diet containing 3%, 6% or 12% cardamom (*w*/*w*) administered in dry form. All experimental diets were isoenergetic and based on the AIN 93 formula (Table 3). The study had a duration of fourteen weeks, and all animals had *ad libitum* access to water and their respective experimental diet. Body weight was measured once a week. Food intake per cage was determined by subtracting the previous day’s diet weight from the diet weight. Daily food intake per mice was estimated by dividing food intake per cage by the number of mice in the cage. Body composition and energy expenditure were evaluated at week 11 and 12, respectively. In the present study, EC seeds obtained from cardamom pods were used in the diet formulation.

At the end of the study, mice were food deprived for six hours prior to the euthanasia; it was performed by administering mice with a lethal dose of sevoflurane. Total blood volume was drawn from the posterior vena cava using a 1 mL syringe with heparin, and plasma was obtained by centrifugation for 10 min at 1500× *g* at 4 °C. Subcutaneous adipose tissue (SAT), visceral adipose tissue (VAT), brown adipose tissue (BAT), liver, and skeletal muscle (soleus and gastrocnemius) were rapidly removed and divided into small samples. Some samples were frozen in liquid nitrogen and stored at −80 °C, and the rest were fixed in ice-cold 4% (*w*/*v*) paraformaldehyde in phosphate buffer saline (PBS) for histological analyses as described below. Brains were excised and frozen at −70 °C until processed, and adrenals were excised and weighed.

### 4.6. Body Composition and Energy Expenditure Measurement

Body composition (lean and fat mass) was evaluated in each mouse using magnetic resonance (EchoMRI; Echo Medical Systems, Houston, TX, USA). Energy expenditure was measured by indirect calorimetry in an Oxymax Lab Animal Monitoring System (CLAMS; Columbus Instruments, Columbus, OH, USA). Animals were individually housed in Plexiglas cages with an open flow system connected to CLAMS for 24 h. Prior to the test, animals were acclimatized for 24 h, fasted for 12 h in the light period and fed during the dark period. Throughout the test, O_2_ consumption (VO_2_, ml/kg/h) and CO_2_ production (VCO_2_, mL/kg/h) were measured sequentially during 90 s. Total O_2_ consumption was calculated and reported as VO_2_. The respiratory exchange ratio (RER) was calculated as the average ratio of produced CO_2_ to O_2_ inhaled (VCO_2_/VO_2_).

### 4.7. Histological Analysis of Liver and Adipose Tissue

Samples of liver and adipose tissue were fixed in PBS-buffered 4% paraformaldehyde, dehydrated, embedded in paraffin and cut into 4 µm slices. Sections were stained with hematoxylin and eosin (H&E) and observed using a Leica DM750 microscope (Leica, Wetzlar, Germany) with a 20× lens. Analysis of the adipocyte area of at least 100 adipocytes per section was performed with the Adiposoft software for ImageJ (ImageJ 1.52K, National Institutes of Health, Bethesda, ML, USA). Two different images per tissue were analyzed after calibration of the software using the scale bar with a length of 100 µm.

### 4.8. Determination of Corticosterone and Triiodothyronine (T_3_) in Plasma

Corticosterone and triiodothyronine (T_3_) concentrations in plasma were determined by enzyme-linked immunosorbent assay (ELISA), using commercial mouse kits from DRG International, Inc. (Springfield Township, NJ, USA) and Thermo Fisher Scientific (Waltham, MA, USA) as described in [54]. Each sample was measured in duplicate.

### 4.9. Determination of Mitochondrial Activity and Lipid Content in Skeletal Muscle and Liver

To analyze mitochondrial activity and lipids content in liver and skeletal muscle, frozen tissues were embedded in optimal cutting temperature (OCT) compound, sectioned with a cryostat (8 μm) and mounted in positively charged slides (Kling-On SFH1103, BIOCARE Medical, Concord, CA, USA). Muscle succinate dehydrogenase (SDH) activity was determined as described in [50]. Briefly, frozen sections were incubated in SDH staining solution (0.55 mM nitro-blue tetrazolium and 0.05 mM sodium succinate) and incubated at 37 °C for 60 min. Afterwards, the slides were washed with deionized water and sequentially dehydrated (2 min/wash) in 30%, 60% and 90% acetone, that was removed with sequential immersion in 60% and 30% acetone and deionized water (2 min/immersion). Digital photographs were taken of each section at 20× magnification as described above, and mitochondrial activity (blue stain) was quantified with ImageJ software. To visualize neutral lipids, frozen tissue sections were stained with 0.5% Oil Red O (ORO) in propylene glycol (Sigma-Aldrich, Burlington MA, USA) as described in [50]. For the quantitative analysis of the ORO staining, the integrated density was determined from the area and the mean gray value was obtained from the measurements taken from each image.

### 4.10. Immunoblotting

Tissues were homogenized at 4 °C in ice-cold RIPA buffer containing PBS, 1% IGEPAL, 0.5% sodium deoxycholate, 0.1% sodium dodecyl sulfate, 1 mM sodium fluoride, 2 mM sodium orthovanadate, and 1 tablet/10 mL of protease inhibitor mixture (Complete Mini, Roche Diagnostics, South San Francisco, CA, USA) in a TissueLyser (Qiagen, Germantown, MD, USA). The samples were incubated on ice for 30 min, centrifuged at 14,500× *g* for 30 min at 4 °C, and the supernatant was transferred to a new tube and stored at −80 °C until use. Protein concentration was determined with the Lowry method [55]. Protein samples (40 µg) were separated on a 10% SDS-polyacrylamide gel and transferred to a polyvinylidene difluoride (PVDF) membranes (Hybond-P, Amersham, GE Healthcare, Chicago, IL, USA) using a wet electroblotting System (Bio-Rad, Hercules, CA, USA). The membranes were blocked for 1 h with 5% non-fat dry milk, and incubated with primary antibodies diluted in blocking solution overnight. Primary antibodies were added as follow: UCP-1 (Abcam, Cambridge, UK, ab155117 dilution 1:2000 BAT), HSL (Abcam, Cambridge, UK, ab45422, dilution 1:2000 SAT), phospho-HSL (Cell signaling, Danvers, MA, USA, 4139, dilution 1:1000, SAT), AMPKa 1/2 (Abcam, Cambridge, UK, ab131512, dilution 1:2000 skeletal muscle), PGC1- alpha (Abcam, Cambridge, UK, ab191838, dilution 1:1000 skeletal muscle), and GADPH (Abcam, Cambridge, UK, ab8245, 1:50,000 BAT and skeletal muscle). Membranes were washed three times with TBS-T for 10 min and then incubated with a horseradish peroxidase-conjugated secondary antibody—goat anti-rabbit 1:10,000, 1:45,000, 1:10,000, 1:7500 and 1:10,000 (Santa Cruz, CA, USA, SC-2357), respectively. Visualization was performed using a chemiluminescent detection reagent (Millipore, MA, USA). Digital images of the membranes were obtained with a ChemiDoc MP densitometer and analyzed using Image Lab 6.1 software (Bio-Rad, Hercules, CA, USA). Results are reported as protein of interest/housekeeping protein ratio or phosphorylated/total protein ratio. A value of 1 was arbitrarily assigned to the control group, which was used as a reference for the other conditions.

### 4.11. Gene Expression of Hypothalamic Peptides

The analysis of hypothalamic arcuate (ARC: NPY and POMC) and paraventricular (PVN) nuclei mRNAs (TRH) expression was performed with real time PCR. PVN (−0.58 to −1.22 mm from bregma) and ARC (−1.22 to −2.46 mm from bregma) [56] were punch-dissected from frozen tissues using a 1 mm diameter sample corer and treated for extraction of total RNA by the guanidine thiocyanate method, as described in [57]. Briefly, guanidinium thiocyanate reagent was added to samples, followed by homogenization using a sonicator. Phenol-chloroform isoamyl alcohol (3/10 of guanidinium thiocyanate reagent volume) was added to the samples, incubated for 15 min and centrifuged at 12,300× *g* for 20 min at 4 °C. The aqueous phase was transferred into a new tube and mixed with isopropanol (1:1 of aqueous phase volume). After an overnight incubation at −20 °C, the RNA was precipitated by centrifugation at 23,400× *g* for 30 min at 4 °C, followed by two washes with cold 75% ethanol. The pellet was vacuum-dried and dissolved in DEPC-treated water. RNA concentration was measured using a biophotometer (Eppendorf, Hamburg, Germany). An amount of 1.5 mg of RNA was retro-transcribed with a high-capacity cDNA reverse transcription kit (Thermo Fisher Scientific, Pleasanton, CA, USA) to obtain cDNA. The mRNA content of NPY, POMC, and pro-TRH was then quantified using Applied Biosystems (Carlsbad, CA, USA) reagents and equipment, comprising TaqMan Universal PCR Master Mix, a StepOne real-time PCR system and TaqMan primers and probe (*Npy*, Mm03038253_m1; *Pomc*, Mm00435874_m1; *Trh*, Mm01182425_g1). Each amplicon was normalized using 18S (Mm03928990_g1) as a reference gene, and each sample was run in duplicate. The amplification program was 40 cycles of 95 °C for 15 s, then 60 °C for 1 min. Changes in different ARC and PVN mRNAs content were evaluated by using the threshold cycle with the ΔCt method; the ΔCt value was obtained from the difference between the number of cycles for each problem gene and that of 18 S to reach the threshold (ΔCt = Ct of unknown genes − Ct 18 S). The control group was used as reference, whose value of fluorescence in arbitrary units was considered as 1, and the percentage of change for each gene examined was obtained by the equation: ΔΔCt = 2 − (ΔCt = Ct Control − Ct experimental group).

### 4.12. Statistical Analyses

Data is expressed as mean ± standard error of the means (S.E.M.). The Shapiro–Wilk normality test was used to check data distribution. All data were analyzed by one-way analysis of variance (ANOVA) followed by Tukey post hoc test using GraphPad Prism 7.0 (GraphPad Software, San Diego, CA, USA) and SigmaPlot 12.3 software (Systat Software Inc., Erkrath, Germany). The differences were considered statistically significant when *p*< 0.05.

## 5. Conclusions

The present study indicates that the intake of cardamom seeds influences the neural circuits that regulate food intake and peripheral energy metabolism. LC-MS and GC-MS analysis shows 11 phenolics and 16 terpenoids in cardamom seeds. Dietary cardamom increases adipose tissue lipolysis and mitochondrial oxidative metabolism in liver and skeletal muscle, leading to increased energy expenditure and lower body fat mass. The effects of cardamom seed intake could be due to parallel central and peripheral actions in the hypothalamus and metabolic organs, or a primarily central effect modulating the HPT and HPA axes and leading to an adjusted hormonal milieu that fine-tunes metabolic activities in peripheral organs. These pre-clinic results pave the way for further research aiming to deepen our understanding of the endocrine and metabolic effects of cardamom, and bolster the use of cardamom seed in the clinical practice as a coadjuvant in the treatment of metabolic diseases.

## Figures and Tables

**Table 1:** Identification of phenolic compounds from a methanolic extract of cardamom seeds by LC-MS analysis (peaks from Figure 1A).

Peak No.	RT	M-H	MS Fragments *	Compound Name and Molecular formula	EC Seeds (mg/100 g)
1	10.70–10.78	153	**109**	protocatechuic acid; 3,4-dihydroxybenzoic acid; (HO)_2_C_6_H_3_CO_2_H	29.69
2	14.00–14.33	153	**109**, 103	gentisic acid; 2,5-dihydroxybenzoic acid; C_7_H_6_O_4_	1.98
3	18.20–18.21	179	**161**, 135	caffeic acid; 3,4-dihydroxybenzeneacrylic acid; (HO)_2_C_6_H_3_CH=CHCO_2_H	26.23
4	18.25–18.35	197	191, 173	syringic acid; 3,5-dimethoxy-4-hydroxybenzoic acid; HOC_6_H_2_(OCH_3_)_2_CO_2_H	36.43
5	19.16–19.22	193	**179**	ferulic acid; 4-hydroxy-3-methoxycinnamic acid; C_10_H_10_O_4_	6.68
6	19.78–18.02	167	**135**, 121	vanillic acid; 4-hydroxy-3-methoxybenzoic acid; HOC_6_H_3_(OCH_3_)CO_2_H	4.28
7	20.8–21.19	353	**190**, 179	5-O-caffeoylquinic acid	14.6
8	22.6–22.8	163	143, **135**, 121	p-coumaric acid; trans-4-hydroxycinnamic acid; HOC_6_H_4_CH=CHCO_2_H	0.44
9	23.08–24.8	397	**191**, 173	sinapoylquinic acid	1.46
10	25.31–27.31	367	173, 161	feruloylquinicacid; 3-*O*-feruloylquinic acid; C_17_H_20_O_9_	0.054
11	31.3–31.34	609	447, **301**	rutin; rutoside; C_27_H_30_O_16_	2.69

* Note: Bold numbers indicate the base peaks in MSn spectra. EC, *Elettaria cardamomum*.

**Table 2:** Identification of compounds present in cardamom seeds by GC-MS analysis (peaks from Figure 1B).

Peak No.	Compound Name and Molecular formula	Mass	Retention Time	EC Seeds (% Area)
1	terpineol cis-α-terpineol; C_10_H_18_O	154	27.17	7.99
2	beta-terpineol; cis-β-terpineol; C_10_H_18_O	154	27.57	1.43
3	linalool; 3,7-dimethylocta-1,6-dien-3-ol; C_10_H_18_O	154	33.66	0.84
4	terpinen-4-ol; (1R)-4-methyl-1-propan-2-ylcyclohex-3-en-1-ol; C_10_H_18_O	154	34.94	0.23
5	α-terpineol; alpha-terpineol; C_10_H_18_O	154	36.22	0.92
6	geraniol; lemanol; C_10_H_18_O	154	37.57	1.05
7	trans-nerolidol; (6E)-3,7,11-trimethyldodeca-1,6,10-trien-3-ol; C_15_H_16_O	222	38.15	0.47
8	α-acorenol; acorenol; C_15_H_26_O	222	39.39	0.52
9	cubebol; (1R,4S,5R,6R,7S,10R)-4,10-dimethyl-7-propan-2-yltricyclo[4.4.0.01,5]decan-4-ol; C_15_H_26_O	222	39.78	0.26
10	isospathulenol; 1aR,7S,7aS,7bR)-1,1,4,7-tetramethyl-2,3,5,6,7a,7b-hexahydro-1aH-cyclopropa[h]azulen-7-ol; C_15_H_24_O	220	41.64	0.26
11	globulol; (1aR,4R,4aR,7R,7aS,7bS)-1,1,4,7-tetramethyl-2,3,4a,5,6,7,7a,7b-octahydro-1aH-cyclopropa[e]azulen-4-oL; C_15_H_26_O	222	42.24	0.75
12	trans-fFarnesol; (2E,6E)-3,7,11-trimethyldodeca-2,6,10-trien-1-ol; C_15_H_26_O	222	43.91	0.62
13	ambrial; 2-(5,5,8a-trimethyl-2-methylidene-3,4,4a,6,7,8-hexahydro-1H-naphthalen-1-yl)acetaldehyde; C_16_H_26_O	234	50.49	5.3
14	eicosane; icosane; C_20_H_42_	282	51.21	0.22
15	costunolide; (3aS,6E,10E,11aR)-6,10-dimethyl-3-methylidene-3a,4,5,8,9,11a-hexahydrocyclodeca[b]furan-2-one; C_15_H_20_O_2_	232	54.43	68.11
16	coronarin; 4-[2-[(1S,4aS,8aS)-5,5,8a-trimethyl-2-methylidene-3,4,4a,6,7,8-hexahydro-1H-naphthalen-1-yl]ethyl]-2-methoxy-2H-furan-5-one; C_21_H_23_O_3_	300	54.85	1.12
17	β-mono palmitin	330	50.59	0.23
18	β-mono stearin	358	56.65	0.39
19	erucylamide; (Z)-docos-13-enamide; C_22_H_43_NO	337	62.71	0.41
20	β-sitosterol; 17-(5-Ethyl-6-methylheptan-2-yl)-10,13-dimethyl-2,3,4,7,8,9,11,12,14, 15,16,17-dodecahydro-1H-cyclopenta[a]phenanthren-3-ol; C_29_H_50_O	414	64.17	0.84

EC, *Elettaria cardamomum*.

**Table 3:** Composition of experimental diets.

		*Elettaria cardamomum* Seed		
%	Control	3%	6%	12%
Proteins	20.30	20.40	20.60	20.90
Carbohydrates	63.70	63.50	63.20	62.70
Fat	16.0	16.10	16.20	16.40
Ingredients (g/kg):				
Casein ^a^	200	196.10	192.20	184.40
Sucrose	100	100	100	100
Maltodextrin	132	132	132	132
Corn starch	397.50	377.58	357.66	317.82
Soy oil	70	67.42	64.84	59.68
Cellulose	50	46.40	42.80	35.60
Vitamin mix ^b^	10	10	10	10
Mineral mix ^c^	35	35	35	35
L-Cysteine ^d^	3	3	3	3
Choline ^d^	2.50	2.5	2.5	2.5
EC seeds		30	60	120
Total	1000	1000	1000	1000

^a^ “Vitamin-free” test, Envigo, Teklad diets, Madison, WI, USA. ^b^ AIN-93, Envigo. ^c^ AIN-93G, Envigo. ^d^ Sigma-Aldrich, Burlington, MA, USA. EC, *Elettaria cardamomum*.

## Data Availability

Not applicable.
